# Seeking a Treatable Cause of Out-of-Hospital Cardiac Arrest during and after Resuscitation

**DOI:** 10.3390/jcm13195804

**Published:** 2024-09-28

**Authors:** Saleem M. Halablab, William Reis, Benjamin S. Abella

**Affiliations:** Department of Emergency Medicine and the Center for Resuscitation Science, Perelman School of Medicine, University of Pennsylvania, Philadelphia, PA 19104, USA; saleem.halablab@pennmedicine.upenn.edu (S.M.H.); william.reis@pennmedicine.upenn.edu (W.R.)

**Keywords:** out-of-hospital cardiac arrest, resuscitation, diagnosis, computed tomography, ultrasonography, electrocardiography, coronary angiography, serum biomarkers

## Abstract

Out-of-hospital cardiac arrest (OHCA) represents a significant global public health burden, characterized by low survival and few established diagnostic tools to guide intervention. OHCA presents with a wide variety of etiologies in a heterogeneous population, posing a clinical challenge to care teams. In this review, we describe evolving research focused on diagnostic approaches to OHCA following resuscitation, including electrocardiography, coronary angiography, computed tomography, ultrasonography, and serologic biomarker assessment. These diagnostic tools have been employed in post-resuscitative efforts for diagnosing ischemic and non-ischemic cardiac, respiratory, neurologic, vascular, traumatic, and metabolic causes of arrest.

## 1. Introduction

Out-of-hospital cardiac arrest (OHCA) remains a leading cause of death in the US and worldwide. While global survival rates from OHCA continue to show modest improvements over recent decades, the disease is still characterized by a high mortality rate; the latest data from the US national OHCA registry, the Cardiac Arrest Registry to Enhance Survival (CARES), indicate a 24% survival rate to hospital admission and 9% survival rate to discharge [1]. The high incidence of OHCA, accompanied by this high mortality rate, emphasizes the enormous need for improved care approaches to OHCA. Increasing prompt OHCA recognition, encouraging bystander cardiopulmonary resuscitation (CPR), and improving emergency medical services’ (EMSs’) response time and preparedness are factors that would increase survival. However, given the marked drop in survival between survival to admission and survival to discharge, the management of patients after resuscitation represents an important area for research and care improvements.

Caring for resuscitated OHCA patients encompasses a complex range of diagnostic tools and multidisciplinary interventions. Increasing efforts have been employed to study interventions aimed at improving outcomes for patients suffering from OHCA, such as targeted temperature management (TTM) and coronary angiography. Several key challenges are inherent in these efforts; the patient population presenting with OHCA to the emergency department (ED) is a highly heterogeneous population of patients with regards to medical and surgical history, age, hereditary disorders, bystander CPR, time to defibrillation, and other factors. Additionally, interventions to reverse treatable causes are likely highly time sensitive, yet in many cases, no attributable cause for the arrest is found, and interventions for those patients are thus limited. This underscores the importance of focusing on post-resuscitation diagnostic modalities that enable the detection of reversible causes or sequelae of the arrest to boost survival rates after the return of spontaneous circulation (ROSC) is achieved.

In this review, we will discuss the available and evolving diagnostic modalities that are helpful in the post-resuscitation assessment of OHCA patients, while linking these modalities to interventions aimed at reversing the respective cause or sequelae of arrest.

## 2. Electrocardiography

Electrocardiography (ECG) is one of the first tools used to assess patients during and after OHCA. During resuscitation efforts, patients are routinely monitored through the continuous acquisition of cardiac electrical activity. Recent studies have attempted to use ECG features during pulseless electrical activity (PEA) to predict ROSC. It has been shown that heart rate on ECG when combined in a logistic regression model with specific electrical waveform characteristics, is a potentially useful prognostic markers of ROSC during PEA [2]. In addition, the role of artificial intelligence (AI) algorithms in assessing for ECG-based markers of ROSC has been explored. Two deep neural networks, trained to differentiate between PEA and pulse-generating rhythms using defibrillator lead pads only, were able to do so with high sensitivity, specificity, and balanced accuracy [3].

After successful resuscitation from OHCA, the acquisition of 12-lead ECGs remains a crucial diagnostic approach. Guidelines from the American Heart Association (AHA) in 2020 recommend routinely obtaining a 12-lead ECG immediately after ROSC and performing a prompt coronary angiography on those patients with evidence of ST elevation on the ECG [4].

An early post-ROSC ECG is important to identify coronary artery disease (CAD) as a reversible cause in OHCA. While this strategy is important for early intervention in cases of myocardial infarction, recent observational data from several studies suggest that an early ECG should be interpreted with caution. Cohort data from Norway suggest that an early ECG post-ROSC is not reliable in identifying patients with indications for an immediate coronary angiography, and it is evident that a considerable subset of patients may suffer from a coronary cause for the OHCA without concerning ischemic changes on the ECG [5]. In the setting of mechanical trauma to the heart during resuscitation, defibrillation and severe metabolic derangements may complicate the interpretation of immediate ECG data. Consequently, some investigations have suggested to either delay ECG acquisition for several minutes post-ROSC, allowing transient injury patterns to dissipate, or to repeat an ECG after some time had passed from an early post-ROSC ECG exhibiting changes consistent with myocardial ischemia [6,7]. However, recent observational data suggest that even a delayed repeat ECG acquisition post-ROSC has a similar accuracy in predicting acute thrombotic coronary occlusion compared to the first early post-ROSC ECG [8]. Interestingly, a study from South Korea that combined ECG, echocardiographic, and biomarker criteria for diagnosing obstructive CAD as a cause for the OHCA, indicates that ECG criteria alone had a more superior diagnostic accuracy than combined criteria [9].

An important opportunity to improve post-arrest ECG interpretation could lie in the use of AI. A trained model was able to successfully diagnose total or near-total coronary occlusion on a post-ROSC ECG, and it was non-inferior to the consensus interpretation of emergency physicians and cardiologists [10].

Finally, the potential role of an ECG in prognosticating patients resuscitated from OHCA has been studied. A post-ROSC ECG has exhibited utility in predicting 30-day mortality: in a multivariate analysis of an international multicenter OHCA cohort, the 30-day mortality was found to be significantly higher in OHCA patients >62 years old, with post-ROSC ECG findings of QRS > 120 ms and ST-segment elevation in >1 segment [11]. In addition, heart rate variability, as measured on ECG during the first day post-ROSC during TTM measures, was able to successfully predict long-term patient outcomes in comatose patients after cardiac arrest [12].

## 3. Coronary Angiography

The role of immediate cardiac catheterization after OHCA remains an ongoing focus of research and controversy. Currently, AHA guidelines recommend an emergent coronary angiography in OHCA patients suspected to have myocardial infarction and accompanying ST-segment elevations on an ECG [4,13]. However, for patients with post-arrest ECG findings that suggest potential ischemia but do not exhibit ST elevations, selecting patients that would benefit from a coronary angiography remains a key challenge in post-resuscitation care. 

Obstructive CAD may trigger OHCA without exhibiting ischemic changes on an ECG, complicating decision making and patient selection for angiography. Earlier observational data examining the use of early cardiac catheterization in comatose patients resuscitated from OHCA without evidence of STEMI on an ECG found significantly decreased mortality [14,15]. A subsequent meta-analysis also supported the use of an early coronary angiography but highlighted the need for randomized trial evidence [16]. This prompted the development of prospective clinical investigations such as the Coronary Angiography after Cardiac Arrest (COACT) trial. In this work, patients were randomized into an immediate versus delayed coronary angiography in OHCA if they presented with initial shockable rhythms, without evidence of ST-elevation myocardial infarction (STEMI) on an ECG. The trial demonstrated no significant difference in survival at 90 days, suggesting that patients without ECG evidence of STEMI findings may not uniformly require immediate angiography [17]. Similar results emerged from additional randomized trials, further highlighting no difference in clinical outcome between an immediate and delayed angiography [18,19,20,21,22].

Whether a subset of patients can be prospectively identified, via either an ECG or clinical factors, who might benefit from an immediate angiography despite the lack of ECG evidence of STEMI, remains an unresolved question [23].

## 4. Computed Tomography

The use of computed tomography (CT) imaging immediately following OHCA resuscitation has varied widely in clinical practice due to the absence of clear guidelines for its use. The European Resuscitation Council and European Society of Intensive Care Medicine Guidelines in 2021 recommended the use of head CT and/or a CT pulmonary angiography in patients without an identifiable cardiac cause for the arrest, and in those presenting with pre-arrest symptoms suggestive of pulmonary or neurological etiology [24]. This is predicated on the notion that CT evaluation may identify treatable underlying causes of arrest, potentially improving the outcomes for patients with OHCA.

Observational studies have evaluated the diagnostic yield and utility of CT imaging following OHCA. These studies highlight the ability of CT to detect a wide range of potential causes and sequelae of OHCA or resuscitation, such as subarachnoid hemorrhage, cerebral edema, pulmonary embolism, pneumothorax, sternal or rib fractures, lacerations findings, among others [24,25,26,27,28,29]. Recent prospective data, employing a standardized protocol for head-to-pelvis CT imaging in patients following OHCA resuscitation, concluded that such a protocol is safe, expedites time-sensitive diagnoses, and provides meaningful clinical information when compared to the standard of care in which clinicians opted for CT imaging at their discretion. These findings were recently confirmed by work from our team in a multihospital cohort [30,31,32,33].

The impact of CT evaluation on the timing of other interventions remains a potential issue; however, one retrospective study suggested that head CT imaging for OHCA patients failed to identify any cases of intracranial hemorrhage, but also resulted in a delay of coronary intervention for those found to have STEMI. While CT practices vary institutionally, the present data concerning the utility of protocolized standardized CT imaging after OHCA resuscitation call for additional prospective investigations to better understand the utility of post-arrest CT evaluation.

## 5. Ultrasonography

Ultrasound is an appealing imaging modality for cardiac arrest resuscitation. It is a mobile, non-invasive, inexpensive, real-time imaging modality that can be potentially utilized during arrest management. While image quality and interpretation are clinician dependent, it is a modality for which emergency medicine and critical care physicians have grown in proficiency over recent years. Ultrasound can be utilized for three major roles within cardiac arrest care: rhythm diagnosis, the evaluation of underlying arrest etiology, and the real-time assessment of CPR performance.

### 5.1. Rhythm Diagnosis

The presenting arrest rhythm is important in guiding subsequent treatment and prognosis. The initial rhythm is included in nearly every model of prognostication, and, in many models, it is the single most consequential predictor of the outcome [34,35]. The arrest rhythm is nearly always derived from an ECG analysis, except in rare cases of fine VF; and in most PEA arrests, a further discrimination of the rhythm can be made using ultrasonography. 

While shockable rhythms almost always present as such on an ECG, ultrasound occasionally reveals an underlying rhythm not detected on the ECG. Although it is rare, it is a known phenomenon that fine VF can present electrocardiographically as asystole or even PEA. Ultrasound discovery of fine ventricular fibrillation (VF) may indicate the responsiveness to defibrillation (despite an evidently non-shockable ECG) and subsequently improves outcome [36,37].

All cases of PEA can be sub-classified as true PEA and pseudo-PEA (pPEA), a state in which organized cardiac activity can be detected despite a pulseless state and PEA electrical rhythm. Pseudo-PEA is more likely to be associated with various reversable outcomes and generally has a higher chance of survival, though it depends on the exact underlying etiology [38,39]. 

### 5.2. Arrest Etiology

#### 5.2.1. Shockable Arrest

ACLS guideline treatment protocols are initially organized around the presenting rhythm. All arrest patients receive CPR, and then patients with shockable rhythms receive defibrillation and specific pharmacologic interventions, while patients with non-shockable rhythms require a further evaluation for potential non-cardiac etiologies. For VF arrests, it is often assumed that the underlying physiology is more likely than not myocardial ischemia [40], although there are other etiologies associated with VF including channelopathies and valvular disease [41]. Ventricular tachycardia (VT) is frequently associated with structural heart disease, including either acute ischemia or electrical activation from previous scarring related to prior ischemia; although VT can also be caused by channelopathies, cardiomyopathies, and other structural defects. Following resuscitation from VF or VT arrest, cardiac ultrasonography may demonstrate regional wall motion abnormalities, which helps confirm the diagnosis of coronary ischemia. The sonographic evidence of wall motion abnormalities, in the setting of shockable arrest, should prompt clinicians to consider a timely angiography for further investigation and/or treatment.

#### 5.2.2. Non-Shockable Arrest

Where echocardiography plays a largely confirmatory role in shockable arrest, it plays a potentially more important diagnostic role in non-shockable arrest, specifically in PEA. PEA and pPEA often both represent heterogeneous underlying disease processes that lack rhythm-specific treatment. The treatment of PEA, in addition to CPR and epinephrine, depends on the underlying physiology. PEA is classically taught to have reversable causes in some cases, many of which may be diagnosed by an echocardiographic investigation, leading to subsequent focused treatment.

Resuscitative ultrasound protocols have been developed to search for sonographic signs of a reversable cause during cardiac arrest. These include the CASA protocol [42], FEER protocol [43], PEA protocol [44], and RUSH exam [45], among others. These strategies likely offer a tradeoff of varying sensitives (through more comprehensive images), at the expense of speed; no head-to-head comparison of these protocols has ever been conducted, and thus the clinician must decide on the extent of the sonographic investigation based on clinical resources and pre-test suspicion for an underlying etiology. 

A comprehensive form of echocardiographic investigation is exemplified by the Motol University approach using an “ABC” checklist [46]. The “A” represents an airway evaluation, which is to say one could use ultrasonography to confirm ETT placement; however, this is rarely carried out in practice. “B” represents a lungs evaluation to assess for tension pneumothorax, pulmonary edema, and other abnormalities. “C” denotes a cardiac evaluation, which is frequently the first and most impactful set of images in various protocols. A subxiphoid view can provide information about pericardial effusion, PE (RV > LV, D sign, McConnell’s sign), MI (wall motion abnormality), and hypovolemia (collapsing/underfilled IVC). In PEA, more so than in any other arrest rhythm, the ability to diagnose the underling etiology in real-time allows for arrest-specific treatment, thus potentially improving the chance of survival.

### 5.3. CPR Quality

Intra-arrest ultrasound also plays an important role in the real-time assessment of the initial arrest outcome, as well as CPR quality. Some studies have suggested that ultrasound may be superior to manual palpation for determining ROSC. Clinicians cannot reliably palpate low systolic pressures, generally taught to be less than 60 mmHg, at the femoral artery. However, ultrasound is more sensitive to flow indicating ROSC. Peak systolic velocities (PSVs) greater than 20 cm/s have an increased accuracy compared to manual palpation [47] and there is evidence to suggest that ultrasound pulse check durations are comparable to manual pulse checks [48].

While ultrasound images may provide important data during CPR, some question whether the CPR quality, and ultimately survival, suffers as a result of attempts to obtain clear images of the chest, potentially increasing CPR pause times. Evidence on this question is limited. 

Regarding the impact of ultrasound on CPR quality, Lien et al., 2023, in a retrospective study of 3300 CPR pauses, showed that using ultrasound during intra-arrest care did not lower the compression fraction; although the ultrasound use reduced the rate of ROSC and failed to improve survival (although it did not worsen it) [49]. Conversely, in a prospective study of 110 pauses, ultrasound during CPR significantly increased the CPR pause length by a mean of 6 s [50]. It is possible that ultrasound use increases CPR pause time, which is a serious consideration given the therapeutic value of minimizing CPR interruptions [51]. 

Regarding the effects of ultrasound on arrest survival*,* a meta-analysis from 2023 that examined ultrasound use during CPR found “no difference in rate of return of spontaneous circulation (RR 0.83, 95% CI 0.24–1.66, *p* = 0.60) (very low certainty of evidence) and a significant decrease in rate of survival to hospital discharge (RR 0.44, 95% CI 0.22–0.88, *p* = 0.02) (very low quality of evidence)” [52]. Contemporary data suggest that ultrasound use has not been associated with improved cardiac arrest outcomes, but given the low quality of data, and the theoretical benefits of ultrasound, more research must be conducted before changing the current use of ultrasound in cardiac arrest care.

### 5.4. Transesophageal Echocardiography

Transesophageal Echocardiography (TEE) is a more invasive modality of ultrasonography that has a number of advantages over transthoracic echocardiography (TTE). TEE does not require access to the chest, which is complicated by ongoing CPR. As a result, TEE can allow for continuous imaging of the heart, unlike TTE, which is usually intermittent, in tandem with CPR and pulse checks. As a result, TEE can be performed during active CPR, which ultimately allows for the real-time continuous visualization of CPR quality. While few emergency physicians are currently trained to interpret TEE images, skilled operators can provide feedback on the “Area of Maximal Compression” (where compressions are having a primary impact on myocardial tissue), Left Ventricular Outflow Tract (LVOT) opening, and heart contractility [53], all of which are potential determinants of survival. Finally, there is evidence that TEE may have a higher sensitivity to identify reversible causes of arrest [54].

### 5.5. Prognostication

While the diagnostic role of ultrasound requires more research, its ability to prognosticate ROSC and survival is more clear, especially in PEA. The distinction between PEA and pPEA is of particular importance when trying to prognosticate survival; a distinction that is best differentiated through ultrasound. A meta-analysis of 11 studies, including 777 PEA patients, found a substantially increased rate of ROSC (RR = 4.35 [95% confidence interval [CI], 2.20–8.63; *p* < 0.00001]) for pPEA compared to true PEA [55]. In addition to predicting ROSC, the discovery of pPEA significantly prognosticates both survival to hospital admission [56], as well as survival to hospital discharge [56]. While the diagnosis of pPEA is not integrated into any widely accepted Termination of Resuscitation (TOR) guidelines, discovering pPEA denotes a significantly better prognosis, of which clinicians should be mindful.

## 6. Serum Biomarkers

Serum biomarkers represent an additional source of information in cardiac arrest management. IV access is nearly always obtained at some point during resuscitation, and therefore blood sampling is both common and practical. A number of commonly ordered laboratory tests and less common biomarkers may have potential diagnostic value during resuscitation and post-arrest care, as described below.

### 6.1. Electrolytes

#### 6.1.1. Potassium

The serum potassium level is a critical data point for resuscitation decision making. Hyperkalemia is a well-described cause of PEA and is especially common in patients with pre-existing renal dysfunction [57]. Other common causes of hyperkalemia include overdose syndromes from medications such as ACE inhibitors, rhabdomyolysis, and tumor lysis syndrome [58]. 

Hypokalemia is most often caused from diuretic use, although it is also caused by diarrhea, DKA, and alcohol use. At levels less than 3 mmol/L [59], which classically cause u-waves, hypokalemia can cause cardiac arrest by hyperpolarizing cardiac membranes, leading to early after depolarizations that devolve to Torsades de Pointes and subsequent VT.

#### 6.1.2. Sodium

Sodium plays an essential role in myocardial action potentials; however, interestingly, sodium derangements are uncommon primary causes of cardiac arrest. Conversely, sodium levels are frequently abnormal after cardiac arrest, and the degree of derangement may predict outcomes in some instances [60,61]. While hyponatremia can represent both volume overload, such as in cases of congestive heart failure, as well as dehydration, the determination of fluid status during resuscitation is probably best made clinically and/or with echocardiography.

#### 6.1.3. Calcium

Calcium handling is essential for both action potential and myofibril contraction physiology. In a retrospective case-control analysis of 770 patients, hypocalcemia was correlated with higher odds of cardiac arrest. Specifically, Ca levels lower than 8.95 mg/dL were associated with a 2.3-fold increase in the odds of SCA comparing to levels higher than 9.55 mg/dL (OR = 2.33, 95% CI: 1.17–4.61) [62]. Hypocalcemia can prolong QT intervals, which is associated with Torsades de Pointes and shockable rhythms. Hypercalcemia does not seem to be a common cause of cardiac arrest [63].

#### 6.1.4. Magnesium

Hypomagnesaemia has been associated with a higher incidence of arrest, perhaps by prolonging the QT interval. In a prospective study of 9820 patients, chronic hypomagnesaemia was associated with an increased risk of arrest [64]. Hypomagnesemia predisposes the myocardium to Premature Ventricular Contractions (PVCs), which can devolve into Torsades de Pointes and subsequently shockable rhythm [65].

#### 6.1.5. Glucose 

Glucose is one of the most immediately available laboratory results during arrest resuscitation and is frequently obtained by EMS via point-of-care testing. Hypoglycemia presents the greater risk for cardiac arrest, compared to hyperglycemia. Profound hypoglycemia (<15 mg/dL) can cause reflex catecholamine release that causes sinus tach that then devolves into AV dissociation, bradycardia, and finally cardiac arrest [66]. In non-arrest situations, hypoglycemia may mimic both coma and stroke.

Hyperglycemia, as an isolated diagnosis, is not known to cause cardiac arrest. However, the sequela of hyperglycemia, particularly DKA, which is associated with other aforementioned electrolyte derangements, can lead to cardiac arrest [67]. Like many other electrolyte abnormalities, glucose derangements are frequently noted on peri-arrest laboratory work, and are associated with poor outcomes, but it is not always possible to diagnose the abnormality as the causal etiology of arrest [68,69].

### 6.2. Liver Biomarkers

The liver is a highly perfused organ, and thus demonstrates a high degree of ischemic injury during cardiac arrest. Prognostically, clotting times (PT/PTT/INR) may inform decisions regarding coagulopathy reversal, especially if hemorrhage is thought to be the cause of cardiac arrest. Conversely, sub-therapeutic clotting times may suggest thrombosis (MI or PE) as the cause of arrest.

Liver damage is both a common and poor prognosticator of cardiac arrest outcomes. A study of 374 post-arrest patients demonstrated that acute liver failure (ALF) (defined as a bilirubin >1.2 mg/dL and an international normalized ratio ≥ 1.5.) was present in 56% of the post-arrest patients, while hypoxic hepatitis (defined as an aminotransferase level >1000 IU/L) developed in 7% of these patients, and 6% of patients developed both conditions. Patients who developed hypoxic hepatitis, but not necessarily acute liver failure, had an increased incidence of poor neurological outcomes in this study [70]. In a separate study (which defined ALF as an INR > 1.5), patients with ALF at admission had substantially increased odds of 28-day mortality (Odds = 10.6, 95% CI 1.36–83.04, *p* = 0.024) [71].

### 6.3. Lactate

Many studies have investigated the role of lactate in cardiac arrest evaluation, given its correlation to hypoperfused states. Lactate is frequently elevated in cardiac arrest patients when tested shortly after resuscitation. Prognostically, a meta-analysis of 23 studies involving 6720 cardiac arrests found that higher lactate concentrations were associated with worse outcomes. Lactate clearance rates provide more predictive prognostic information than single-point measurements [72,73].

### 6.4. Cardiac Biomarkers

Multiple biomarkers used in clinical practice may help diagnose the cardiac etiology of arrest. 

#### 6.4.1. Troponin

Troponin levels are frequently elevated after cardiac arrest. While elevated troponin levels are commonly associated with myocardial ischemia, classically suggesting the need for early angiography, multiple studies have failed to find a troponin cut off that predicts the need for angiography in the cardiac arrest population [74,75]. Physiologically, this is likely due to troponin elevations inherent in arrest-related ischemia, as well as iatrogenic myocardial injury from CPR and defibrillation. The inability of troponin levels to aptly discriminate the need for PCI may also be because of a fairly well-established element of ischemic signs of CAD dissociation in cardiac arrest. For example, it has been demonstrated that around 30% of non-STEMI arrests are found to have significant CAD, requiring PCI [17]; conversely, there are some patients with STE on a post-ROSC ECG who do not have an underlying coronary obstruction [6].

#### 6.4.2. Brain Natriuretic Peptide

Unlike troponin levels, which are commonly elevated by peri-arrest processes, brain natriuretic peptide (BNP) levels may provide more useful information. In a smaller trial of 70 patients, Hwang et al. demonstrated two useful characteristics of BNP levels. First, it has some ability to discriminate between cardiac etiologies and non-cardiac etiologies (odds 2.1 of cardiac etiology with BNP ≥ 100 pg/mL). Second, BNP levels do not seem to vary after receiving CPR, which implies that CPR and the arrest itself do not elevate the BNP level [76]. BNP levels are associated with cardiac etiology, which is unsurprising considering the significant association with BNP levels, heart failure, and the risk of SCD [77].

#### 6.4.3. D-Dimer

One of the significant etiologies of arrest, particularly PEA, is massive pulmonary embolism (PE). Difficult to diagnose clinically, PEs classically present without physical exam findings and frequently do not have ECG findings. PEs are definitively diagnosed with a chest CT evaluation, but this first requires resuscitation and stabilization. A D-dimer assessment provides indirect evidence of clotting and thus potentially thromboembolic events. D-dimer elevations are frequently falsely elevated by advanced age, malignancy, and chest trauma [78], which are similarly risk factors for cardiac arrest [79]; thus, dimers are at risk of being falsely elevated in cardiac arrest patients. Similar to troponin levels, CPR can iatrogenically elevate D-dimer levels. Asano et al. demonstrated that the D-dimer levels were elevated in 97.7% of arrest patients, and that the D-dimer levels correlated with the duration of CPR [80].

### 6.5. Infection Biomarkers

Complete blood count, different microbial cultures, and procalcitonin are important indicators of ongoing infectious processes. Interpreting these results may be challenging given that infection may present as a cause or sequelae of cardiac arrest.

#### 6.5.1. White Blood Cell Count

Nearly universally obtained after cardiac arrest, the Compete Blood Count (CBC) provides useful diagnostic and prognostic information after cardiac arrest. As cardiac arrest is a profoundly inflammatory state (due to CPR trauma, ischemia-reperfusion injury, etc.) it frequently results in leukocytosis. Interestingly, the most common admission white count is in the normal range (about 60% of post-arrest patients); however, leukocytosis (about 30% of patients) is a common finding [81]. Like many post-arrest derangements, worsening leukocytosis, especially neutrophilia and monocytosis, is associated with worse outcomes [81].

#### 6.5.2. Blood Cultures

Blood cultures are a useful adjunct to help differentiate true infection from reactive leukocytosis. Rech et al., in a retrospective single-center study, found a true bacteremia rate of about 16% in post-cardiac arrest patients; and bacteremic patients had worse mortality [82]. Retrospective single-center studies found a higher rate of bacteremia in OHCA patients (38% and 46.5%), and interestingly found that bacteremia patients most often presented with non-shockable arrest [83,84]. It is worth noting that the presence of bacteremia presents its own diagnostic challenges. It is known that sepsis can, itself, cause cardiac arrest in nearly 10% of septic patients [85]. Conversely, it is well-documented that sepsis is a common consequence of cardiac arrests with estimates ranging from 13 to 27% [86]. The key to differentiating sepsis as cause or consequence lies in history, lab timing, and clinical reasoning.

### 6.6. Toxicology Screening

Substance abuse remains an important cause of OHCA. A study by Rittenberger et al. found that drug overdose was the cause of nearly 15% of arrests, and the contributing drugs were most often opiates, benzodiazepines, or both; with some contribution from cocaine, methadone, marijuana, and ethanol [87]. Interestingly, overdose patients in this study were less likely to present in PEA (odds 0.58 [0.31–1.07] *p* = 0.08). A recent study Stampe et al. evaluated the utility of creating a toxicological profile using mass spectrometry for cardiac arrest patients. The authors found that 82% had at least one drug detected at SCA, polypharmacy was common (19%), and commonly abused drugs were encountered in 16% of SCA, but ultimately the clinical utility of complex toxicological screen was low [88].

## 7. Knowledge Gaps and Future Directions

The discussed modalities are important to aid clinical teams in the diagnosis of post-arrest etiologies and the potential treatment of any treatable causes. ECG, CT imaging, and serologic testing are widely available in most EDs and are regularly utilized in post-arrest diagnostics. However, the broader implementation of other diagnostic tools faces several limitations. In many resource-limited settings, access to trained sonographers, particularly during off-hours or holidays, may be limited. This challenge is even more pronounced for TEE, which requires specialized training to both perform and interpret images. Similarly, some centers may not have a cardiac catheterization team available 24/7 to perform a coronary angiography after arrest. Highlighting the importance of these diagnostic modalities in post-arrest care could drive efforts to increase the availability of these resources in these areas.

While this review has focused on novel research on diagnostic modalities available for post-resuscitation care in OHCA patients, significant gaps in its knowledge remain, particularly regarding post-resuscitation diagnosis, survival, and prognostication in OHCA. These topics will require additional investigational work. International resuscitation guidelines have been insufficiently clear regarding post-arrest diagnostic methods to detect treatable arrest etiology or sequelae. In the pre-hospital setting, current guidelines and research efforts have primarily focused on basic and advanced cardiopulmonary support, with limited evidence on the use of diagnostic modalities by EMS to accelerate the identification of critical or reversible causes of OHCA. Training EMS personnel in the use of point-of-care ultrasound (POCUS) after ROSC could enhance early post-arrest evaluation and care. Two studies have reported the successful integration of POCUS into EMS care [89,90]. Furthermore, while EMS personnel can perform limited ECG interpretation, more complex or subtle ECG changes often require specialized training. This presents an opportunity to test and integrate available AI models to assist in rapid ECG interpretation in the pre-hospital setting. Additionally, there are a notable lack of data on neuroprognostication following survival from OHCA, which remains a critical area for further investigation—a subject under clinical investigation by the RECOVER program [91]. Neuroprognostication is essential for guiding clinical decisions about ongoing care or transitioning to comfort measures. Current tools like EEG, SSEP, and biomarkers offer limited predictive accuracy. The heterogeneity of OHCA patients further complicates prognostication, underscoring the need for more personalized approaches. Future research should prioritize refining neuroprognostic models, identifying novel biomarkers, and improving imaging techniques to reduce uncertainty in post-arrest care.

## 8. Conclusions

**Figure 1:**
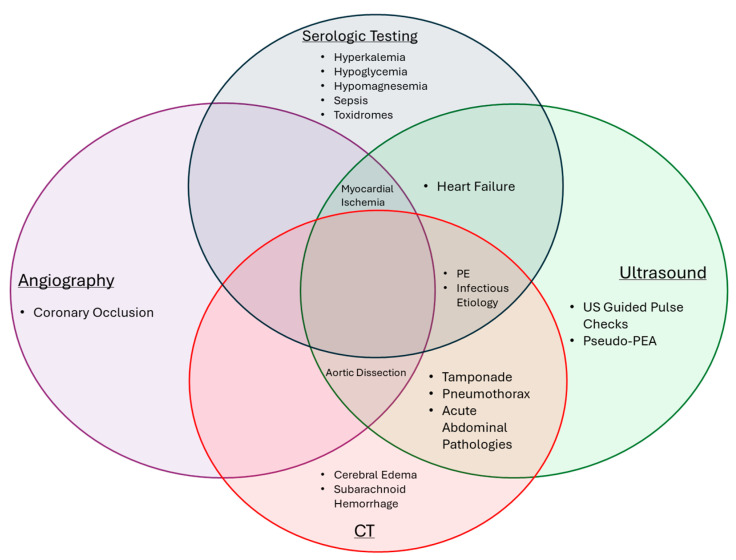
Diagnoses made possible by various investigational modalities. Investigational modalities are represented by large circles, with the causes of cardiac arrest that they can identify in the area of the circles. CT, computed tomography; PE, pulmonary embolism; US, ultrasonography; and PEA, pulseless electrical activity.

OHCA is a complex syndrome that requires an elaborate chain of collaboration between bystanders, EMS, and hospital teams. High-quality resuscitation, post-resuscitation diagnosis, and subsequent procedural or medical interventions remain the cornerstone elements to the care of OHCA patients. A combination of serological testing, ultrasonography, CT imaging, and angiography provide essential data points for diagnosing and treating the underlying causes of cardiac arrest (see Figure 1 for a modality centric summary and Table 1 for a diagnosis centric summary). These elements require further research and development to better understand modalities that help in post-ROSC diagnosis and populations that would benefit from available interventions. There remains great potential to improve post-resuscitation care in OHCA, given the consistently high mortality worldwide. In this review, we focused on evolving evidence and research tailored around important diagnostic modalities used in post-resuscitation care, and we look forward to further investigations that will better guide post-arrest care for OHCA and improve outcomes.

## Figures and Tables

**Table 1:** Adjunctive modalities to evaluate the AHA reversable causes of cardiac arrest.

AHA H’s & T’s	Definitive Diagnostic Modality	Useful Adjunctive Investigations	Useful Serum Testing
Hypovolemia	N/A	IVC ultrasound FAST exam	Coagulation testing BMP
Hypoxia	Arterial blood gas	Pulse oximetry End-tidal CO_2_ CTA chest Chest xray Lung ultrasound (B-lines, lung sliding)	Troponin BNP
Hydrogen ion (acidosis)	Blood Gas	N/A	BMP Lactate Serum osmolarity Tox screening
Hypo-/hyperkalemia	Serum Potassium	ECG	BMP
Hypothermia	Vitals	Bladder temperature CT head	BMP CBC TSH Tox screen
Tension pneumothorax	Chest CT	Chest xray Lung ultrasound (lung sliding)	Coagulation testing ABG
Tamponade, cardiac	Cardiac Echocardiography	CT chest ECG	BMP CBC Coagulation testing
Toxins	Toxicology Screening	ECG	BMP CBC Serum osmolarity Salicylate and acetaminophen level
Thrombosis, pulmonary	Chest Angiography	Ultrasound (right heart strain, McConells sign) ECG	Troponin (pre-CPR) BNP D-Dimer (select patients)
Thrombosis, coronary	Cardiac Catheterization	ECG Cardiac Echocardiography CT Coronary Angiography	Troponin (pre-CPR) BNP Coagulation testing

N/A, not applicable; IVC, inferior vena cava; FAST, Focused Assessment with Sonography in Trauma; BMP, basic metabolic panel; CTA, computed tomography angiography; BNP, brain natriuretic peptide; ECG, electrocardiography; CT, computed tomography; CBC, complete blood count; TSH, thyroid-stimulating hormone; and ABG, arterial blood gas.
